# From Negative Duplex to Phlegmasia in Minutes: Bedside POCUS Identifies Rapid Thrombosis Unmasking Underlying May–Thurner Syndrome

**DOI:** 10.1002/jcu.70268

**Published:** 2026-05-14

**Authors:** Anthony Unger

**Affiliations:** ^1^ Department of Emergency Medicine UPMC Hamot Erie Pennsylvania USA

## Abstract

May–Thurner syndrome occurs from an anatomical variant in which the left common iliac vein is compressed against the lumbar spine by the overlying right common iliac artery. This often leads to chronic venous stasis predisposing individuals to left‐sided iliofemoral deep venous thrombosis (DVT). Progression to phlegmasia cerulea dolens (PCD) is rare, but potentially limb‐threatening. Here we describe rapid formation of DVT leading to PCD first discovered during a bedside point‐of‐care ultrasound (POCUS). A 50‐year‐old female presented to the emergency department (ED) with left leg swelling following a recent hospitalization for ulcerative colitis flare with rectal bleeding. In hospital she was treated with corticosteroids and had anticoagulation medication held due to active bleeding. On initial presentation to the ED, a comprehensive duplex exam performed by a vascular sonography technician was negative for DVT, as were the physical exam findings. As there was no concern for DVT, a POCUS was performed in an educational context, with the intention of demonstrating consistent imaging findings. However, POCUS performed just 14 min after completion of the duplex exam identified a rapidly evolving thrombus. The patient's leg pain and edema also progressed with discoloration and paresthesia prompting computed tomography angiography (CTA) imaging and vascular consultation for developing phlegmasia. She was taken emergently to the operating room and was subsequently found to have underlying May–Thurner Syndrome (MTS). The patient required a second vascular intervention and was discharged with a complete recovery. This case highlights the critical role of POCUS in detecting evolving vascular emergencies as it changed the patient's treatment plan, expedited diagnosis, and resulted in successful endovascular interventions.

## Graphical abstract

Point‐of‐care‐ultrasound revealed an evolving acute noncompressible deep vein thrombosis (asterisks) in the left lower extremity veins (right panel) that was not evident on duplex ultrasound 14 min prior (left panel; asterisks demonstrate compressed veins). The patient was treated emergently and found to have May–Thurner syndrome.

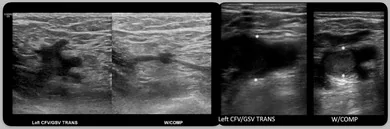

## 1 Introduction

Deep venous thrombus (DVT) is a common vascular condition resulting from the formation of a blood clot within the venous system. DVT typically occurs in the lower extremities and can lead to significant morbidity and mortality if not promptly diagnosed and treated. Phlegmasia cerulea dolens (PCD) is an uncommon vascular emergency, potentially created by the progression of a DVT. The complete or near‐total occlusion of the venous outflow system leads to significant edema that subsequently compresses the arteries and causes the bluish discoloration associated with limb ischemia and potential tissue loss (Chinsakchai et al. 2010; Gardella and Faulk 2022; Galanakis et al. 2021).

May–Thurner syndrome (MTS) refers to the extrinsic compression of the left common iliac vein by the overlying right common iliac artery against the lumbar spine. This often leads to chronic venous stasis and thrombosis. While MTS may remain asymptomatic for years, it is increasingly recognized as an underlying etiology in young or middle‐aged women presenting with unprovoked or recurrent left‐sided DVT (Ahmed et al. 2019).

DVT is usually formally diagnosed by duplex ultrasound performed by a radiologist or vascular technician. Point‐of‐care ultrasound (POCUS) in the Emergency Department (ED) can also identify suspected DVT (Varrias et al. 2021; Spampinato et al. 2024). Cases of suspected DVT identified by POCUS are typically confirmed by duplex ultrasound. A potential advantage of POCUS is that it allows immediate assessment of the patient at the bedside and, as shown in the current case, can be an essential tool to detect dynamic and rapidly progressing pathology.

This report describes a unique case of POCUS providing real‐time visualization of an evolving thrombus just 14 min after a negative formal duplex ultrasound study. POCUS unexpectedly detected a massive, limb‐ and life‐threatening thrombus formation that quickly progressed to PCD in a patient that did not appear to have lower limb thrombus based on physical and duplex imaging. Subsequent computed tomography angiography (CTA) and intraoperative imaging led to the discovery of underlying MTS. In this case, real‐time POCUS findings initiated the rapid response that led to successful vascular interventions and a complete recovery for the patient.

## 2 Case Presentation

A 50‐year‐old female with a history of anemia and ulcerative colitis (UC) presented to the ED with a complaint of progressive left lower extremity swelling beginning that morning. She had been discharged the previous day following a brief two‐day inpatient treatment for a UC flare with rectal bleeding. While admitted, DVT prophylaxis was held to prevent any further bleeding or progression of her baseline anemia. She also received treatment with corticosteroids.

Her presenting chief complaint in the ED was tightness and swelling in her left leg beginning that morning. She denied trauma, prior thromboembolism, chest pain, or shortness of breath. Physical examination revealed subtle edema in the left leg without focal tenderness or discoloration. Both limbs were warm and well‐perfused with preserved distal pulses and sensation.

**FIGURE 1:**
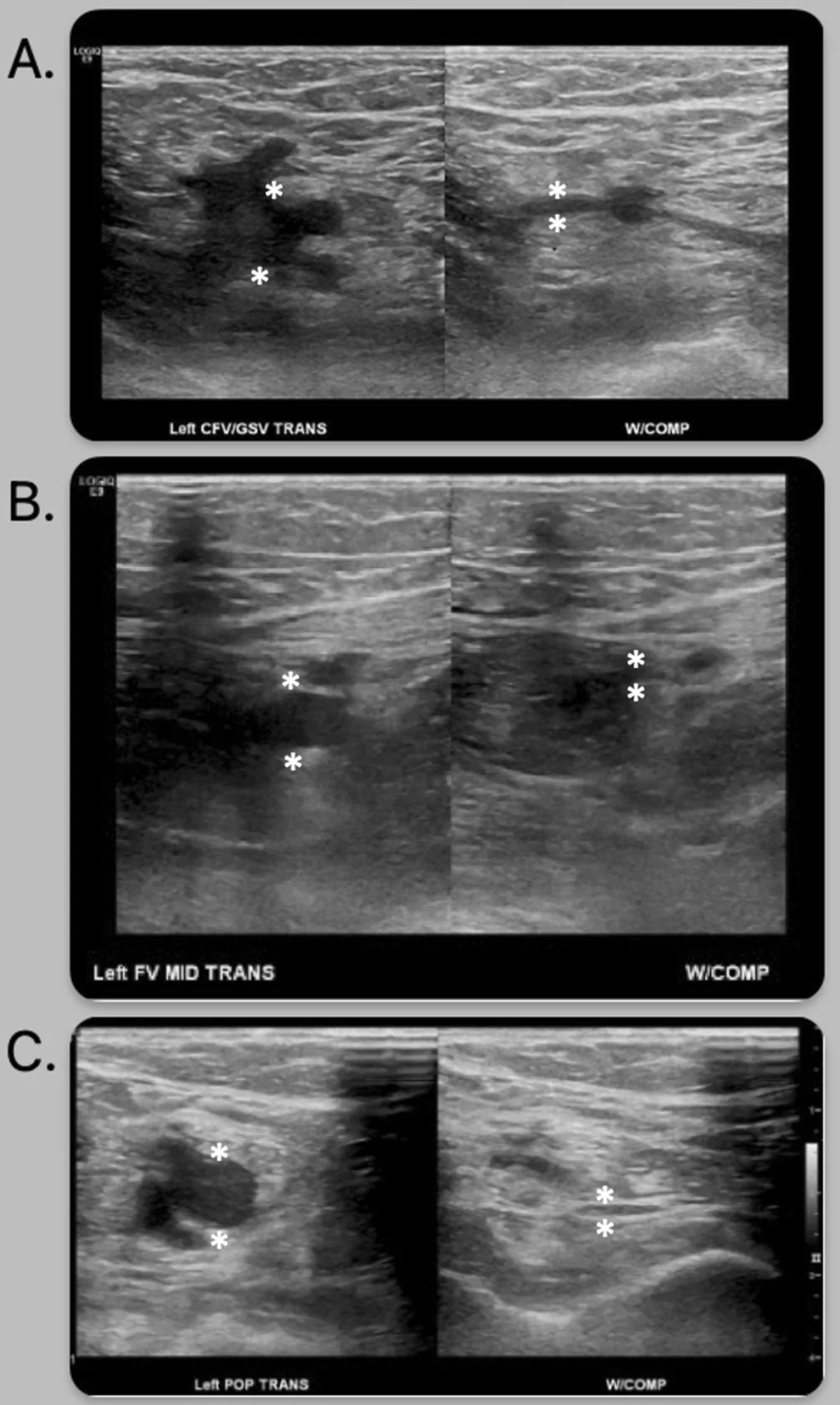
Formal duplex ultrasound without evidence of a deep vein thrombosis in the left lower extremity involving collapsable veins. (A) the left common femoral vein; (B) the left femoral vein; (C) the left popliteal vein. Asterisks indicate each side of the vessel wall being evaluated and show complete collapsibility (right panels) indicative of a negative study.

Initial laboratory studies were notable for chronic anemia and mild thrombocytopenia. Coagulation studies were within normal limits. An experienced vascular sonography technician performed lower extremity duplex ultrasound that was negative for acute DVT (Figure 1). Rouleaux formation was noted throughout the left‐sided venous system. The study included normal respiratory phase and calf augmentation to exclude a proximal thrombus. The sonography technician completed the study with the last image of calf augmentation recorded at 12:30 p.m. (Figure 3a).

While test results and discharge plans were being considered by the ED team, the ED ultrasound education team, led by a POCUS‐credentialled attending and EM ultrasound fellow, reviewed the duplex exam. Given the negative imaging and physical findings, they initiated a bedside POCUS to educate the emergency medicine resident and medical student in attendance. The goal was to demonstrate a negative DVT POCUS study.

**FIGURE 2:**
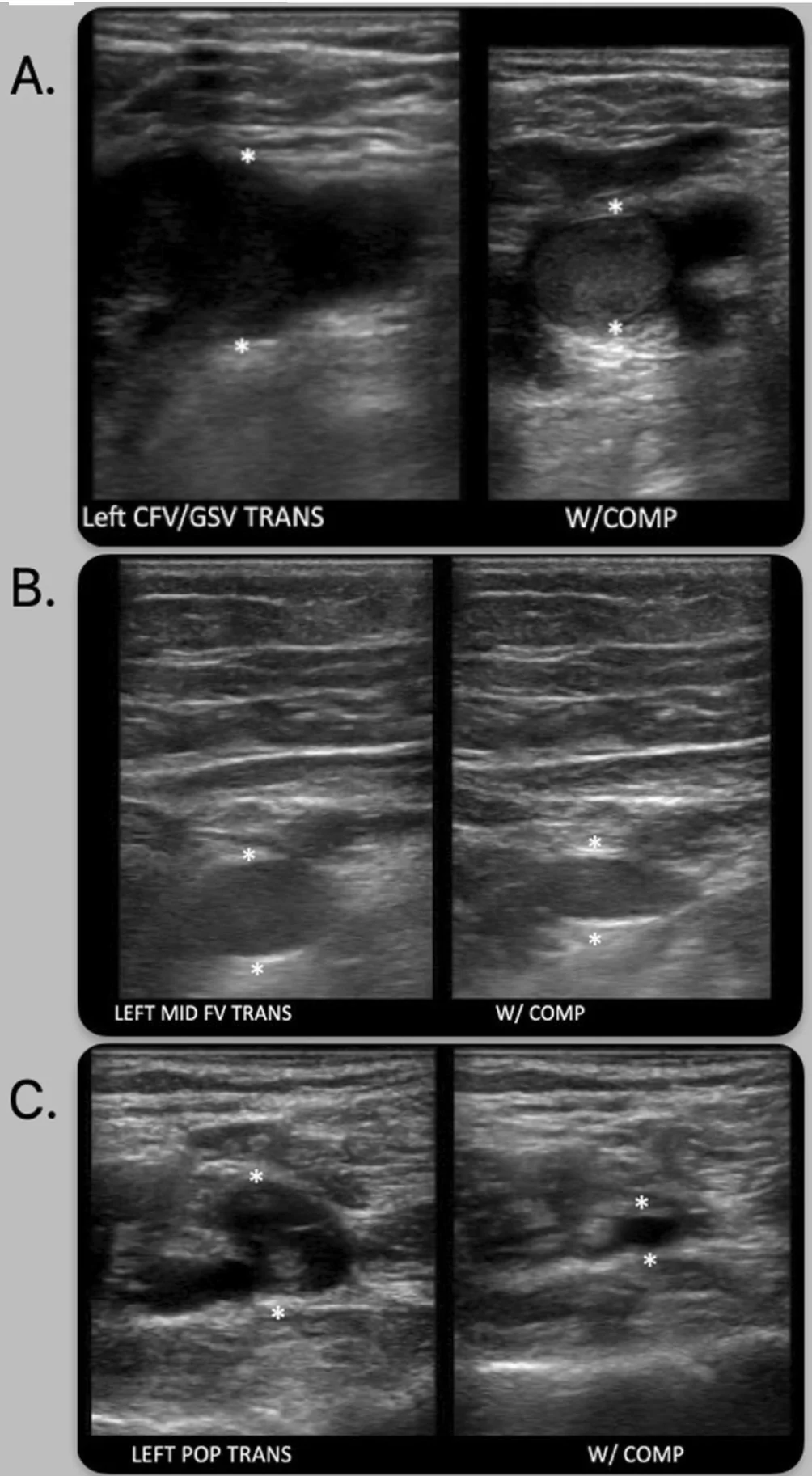
A point‐of‐care‐ultrasound (POCUS) revealed acute noncompressible deep vein thrombosis in the left lower extremity veins. (A) the left common femoral vein; (B) the left femoral vein; (C) the left popliteal vein. Asterisks indicate each side of the vessel wall being evaluated and show noncollapsible veins (right panels) indicative of a positive study.

**FIGURE 3:**
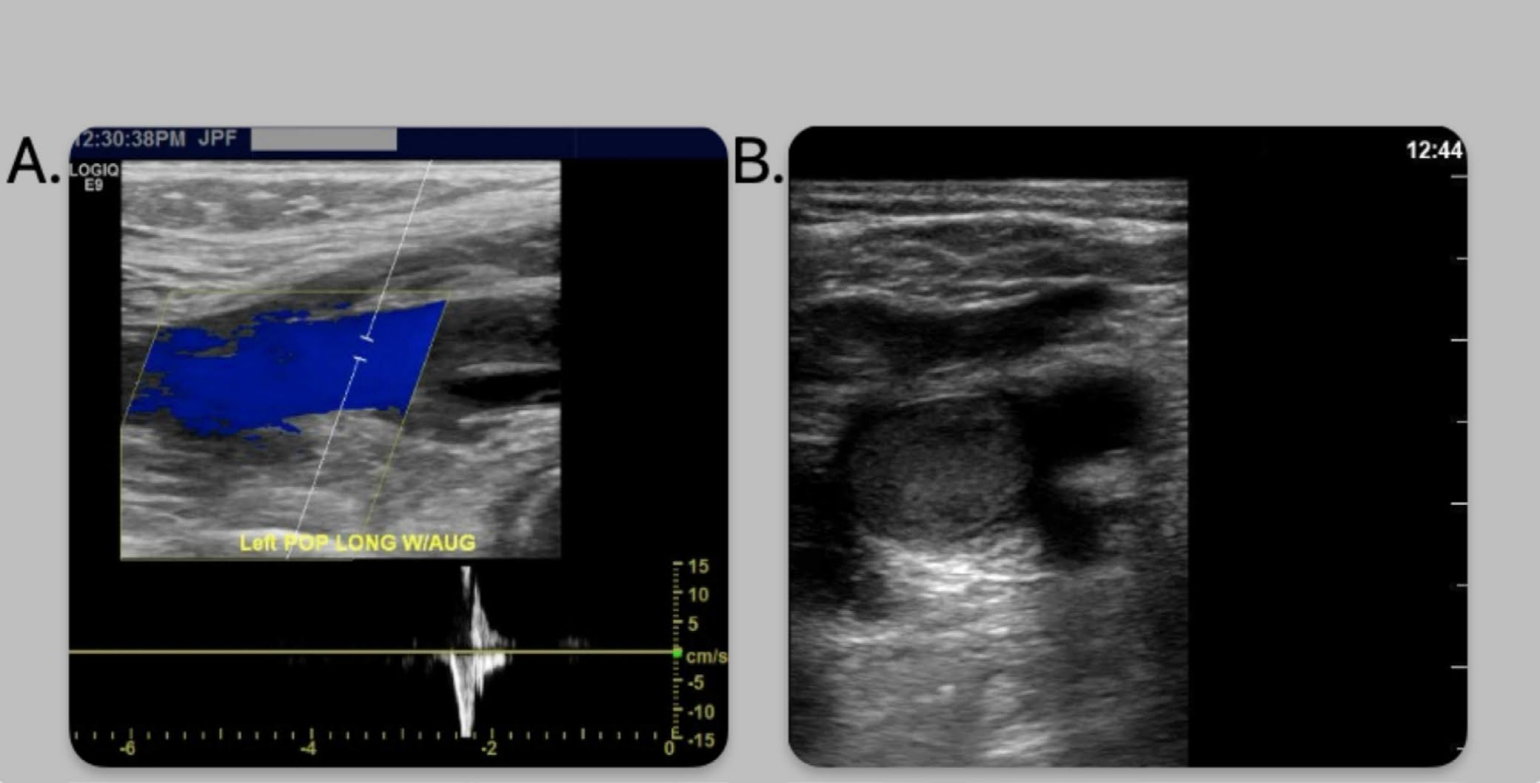
Time gap between sonographic exams (A) the last imagine obtained from the formal duplex at 12:30 p.m. of left calf augmentation with reassuring venous wave form (B) the first imagine obtained from the POCUS exam at 12:44 p.m. of noncompressible left common femoral vein.

However, when the first POCUS images were obtained, they immediately revealed a large hypoechoic structure within the saphenofemoral junction and distal noncompressible common femoral vein, femoral vein, and the popliteal vein (Figure 2). The first POCUS images were recorded at 12:44 p.m., just 14 min after the negative duplex exam (Figure 3b), highlighting how quickly the changes occurred. The POCUS findings raised urgent concern for a rapidly evolving thrombus not detected on the formal duplex exam. Coinciding with the POCUS exam, the patient reported worsening discomfort and upon completion of the exam, she noted an onset of numbness extending from her foot to her calf with difficulty moving her toes. Physical exam revealed intact distal pulses but now demonstrated a dusky, bluish‐red discoloration suggestive of early PCD. This change of physical exam was noted at 12:52 p.m., 22 min after the initial duplex exam was performed.

Vascular surgery was consulted and an emergent computed tomography angiography (CTA) was ordered to rule out arterial contributions given the paradoxical negative formal duplex and POCUS exams. Vascular surgery evaluated the patient as she returned from CTA and confirmed concern for early PCD. The CTA revealed patent arteries with suspicion for extensive lower extremity thrombus. The patient was started on intravenous unfractionated heparin. She was taken emergently to the operating room that afternoon where she underwent left iliofemoral venogram, suction thrombectomy, and balloon angioplasty of the left common iliac vein.

**FIGURE 4:**
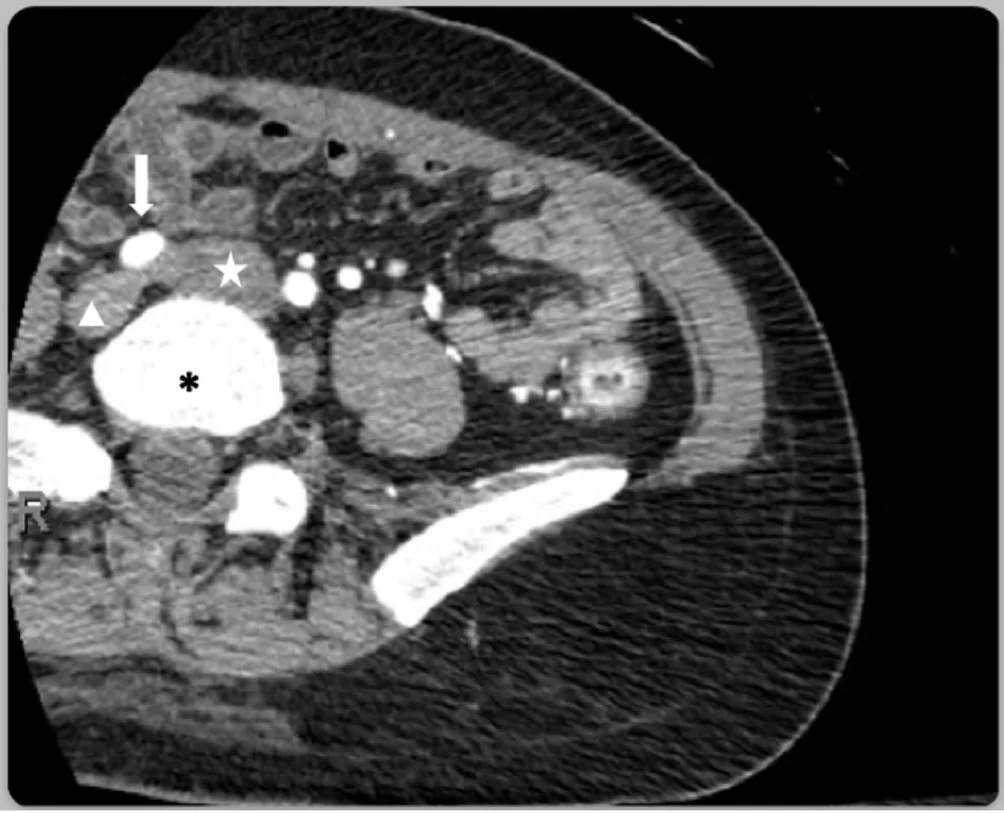
Computed tomography angiography (CTA) performed on the patient. The distention of the left common iliac vein (star) from decreased venous return is apparent when compared to the right common iliac vein (triangle). This image did not directly show the compression of the left common iliac vein (star) by the right common iliac artery (arrow) against the spine (asterisk) as the image did not extend proximally enough.

Following the procedure, her sensory and motor deficits resolved. During review of intraoperative imaging, significant stenosis consistent with May–Thurner Syndrome was observed. This was not evident on the CTA due to scan imaging starting distal to the compression (Figure 4). She returned to the OR the following day for placement of a left external iliac vein stent. The procedures were successful, and she remained asymptomatic.

Hematology was consulted, and the DVT was deemed provoked, with contributing factors including recent hospitalization without DVT prophylaxis, active UC, corticosteroid use, and May–Thurner anatomy. She was transitioned from heparin to apixaban for outpatient anticoagulation and discharged home 2 days after stent placement with vascular surgery and hematology follow‐up. She remains anticoagulated and asymptomatic on her current regimen of apixaban twice daily.

## 3 Discussion

This case highlights the critical role of bedside POCUS in rapidly identifying clinically significant thrombotic progression. Using POCUS, we expedited the diagnosis of an extensive DVT that was undetected 14 min earlier on formal duplex ultrasound. The DVT evolved into PCD, prompting emergent consult and treatment by vascular surgery. Intraoperative imaging subsequently revealed the patient had unrecognized MTS.

This case is unique in that a novel change in sonographic findings was detected within 14 min of a negative duplex exam. The comprehensive duplex exam was performed by a credentialed vascular lab sonography technician with several years of experience and reviewed by an experienced, credentialed vascular surgeon. In addition, the POCUS‐credentialled EM team members also reviewed the duplex images and concurred there was no evidence of thrombus. There was no suggestion of ambiguity in the interpretation of the duplex results. Thus, the duplex exam was likely accurate at the time of imaging, as there were no signs of lower extremity clotting.

The only finding noted from the duplex exam was rouleaux formation, an aggregation of red blood cells resembling stacked coins. Unlike with a DVT, the vessel remains compressible and resolves with augmentation. Rouleaux formation is a common finding and typically has no clinical impact. However, it can be indicative of underlying low‐flow, hyperviscous conditions or a proximal obstruction (Hercz et al. 2024).

On the patient's duplex exam, she had normal respiratory variation of venous return and normal calf augmentation (Figure 1a). These assessments are two indirect ways to exclude a proximal thrombus based on nonobstructed venous flow. Given these normal flow assessments, the nonspecific rouleaux formation was not viewed as a concerning finding. In retrospect, the formation was likely a harbinger of the thrombotic cascade about to ensue.

There were several factors that contributed to the formation of the extensive thrombus. The patient's undiagnosed underlying MTS predisposed her to left sided venous pathology that was likely accelerated by her recent hospitalization for UC flare with rectal bleeding, a sign of underlying endothelial injury. Her 2‐day hospitalization and immobilization also created a state of venous stasis. In addition, the patient's UC treatment consisted of corticosteroids, which increased her blood viscosity. The simultaneous presence of vascular injury, venous stasis, and hypercoagulability increases the risk for clot formation, as described in Virchow's triad (Varrias et al. 2021; Spampinato et al. 2024; Kushner et al. 2024; Blanco and Volpicelli 2016).

This alignment of pro‐thrombotic factors paired with her MTS is believed to be the cause of her rapid progression from a negative duplex to PCD. However, a limitation of this case is that the cause and exact timing of the thrombus cannot be determined.

The patient's transformation from mild edema with a negative radiology‐performed ultrasound to a large, occlusive thrombus detected on ED POCUS within 14 min underscores the dynamic nature of thrombosis in high‐risk individuals. POCUS rarely captures the rapid formation of a thrombus. Other advanced imaging techniques, such as intravenous ultrasound (IVUS) provide real‐time assessments that support the dynamic nature of thrombotic formation. IVUS‐ based studies have demonstrated that acute thrombi may appear as hypoechoic, poorly organized structures with sudden venous flow compromise, demonstrating rapid, clinically significant thrombotic progression over short intervals (Corvino et al. 2025), similar to what we observed.

The patient's continued rapid deterioration and progression to PCD while awaiting CTA and vascular intervention further demonstrates the value of immediate bedside imaging in directing timely care. PCD represents the extreme end of the DVT spectrum. It is characterized by massive iliofemoral thrombosis with near‐total to complete venous obstruction. The decreased venous return drives increasing capillary hydrostatic pressure and results in extensive edema. This edema then increases limb compartment pressures and leads to arterial compression. The subsequent arterial inflow obstruction causes limb ischemia and is associated with high rates of limb loss and mortality if not rapidly treated (Galanakis et al. 2021; Ahmed et al. 2019).

MTS is present in an estimated 2%–5% of the population and contributes to 20%–30% of left‐sided DVTs; however, it is frequently missed due to nonspecific presentations (Ahmed et al. 2019; Spampinato et al. 2024). POCUS has proved to be uniquely beneficial in other cases of MTS. In one case, a patient with suspected lymphadenopathy, POCUS was intended to visualize the lymph nodes but instead revealed a thrombus that was confirmed by duplex ultrasound (Belkin et al. 2021). The patient had no physical signs of DVT and MTS was subsequently revealed on CT imaging. As in our case, POCUS identified the thrombus in time for rapid and successful treatment with complete recovery.

In another case, a patient presented with symptoms suggestive of DVT, but initial duplex ultrasound was negative. However, a CT venogram (CTV) identified a thrombus in the left common iliac vein and revealed vessel compression consistent with MTS. POCUS was used subsequently to visualize the vessels, and the exam confirmed the presence of the thrombus and May‐Thurner anatomy (Brown et al. 2022). The authors suggested potential benefits of using a lower abdominal POCUS exam as a first line assessment in patients with suspected left lower extremity DVT and negative duplex ultrasound to limit risks associated with radiation exposure and contrast dye injection associated with CTV.

Overall, POCUS in the ED setting has been shown to be sensitive and specific for detecting DVT, particularly when done by ED physicians with training and experience in POCUS (Varrias et al. 2021; Spampinato et al. 2024). As seen in our case, POCUS can also identify a rapidly evolving thrombus and expedite successful patient outcomes.

To our knowledge, this is a novel case of rapid thrombus formation detected by POCUS after negative formal duplex imaging. The patient had significant risk factors coupled with her underlying anatomy, creating the pathophysiological setting for progression.

## 4 Conclusion

Bedside POCUS plays a critical role in early detection and urgent vascular intervention in dynamic pathology. Clinicians should remain alert to evolving thrombotic syndromes, especially in the setting of multiple predisposing risk factors. MTS should be considered in patients, particularly women, with unexplained or rapidly progressive left‐sided DVT symptoms.

## Funding

The author has nothing to report.

## Ethics Statement

This study did not require review by our institutional review board as it is a description of a single case without identifying information.

## Consent

Written consent was obtained from the patient before the publication of this case report.

## Conflicts of Interest

The author declare no conflicts of interest.

## Data Availability

The data that support the findings of this study are available on request from the corresponding author. The data are not publicly available due to privacy or ethical restrictions.
